# Prevention of lung-to-lung aspiration during emergency thoracic surgery: an experimental study

**DOI:** 10.1186/s13017-015-0009-6

**Published:** 2015-03-04

**Authors:** Jin-Young Hwang, Jiseok Baik, Sahngun Francis Nahm, Dongjin Kim, Young-Tae Jeon, Jinhee Kim, Seongjoo Park, Sunghee Han

**Affiliations:** Department of Anesthesiology and Pain Medicine, Borame Medical Center, Seoul National University, College of Medicine, Boramae-ro 5-gil, Dongjak-gu, Seoul, Kyoneggido 156-707 South Korea; Department of Anesthesiology and Pain Medicine, Pusan National University Hospital, Biomedical Research Institute, Pusan National University, School of Medicine, 179 Gudeok-ro, Seo-Gu, Busan, 602-739 South Korea; Department of Anesthesiology and Pain Medicine, Seoul National University Bundang Hospital, Seoul National University, College of Medicine, 300 Gumidong Bundanggu, Seongnamsi, Kyoneggido 463-707 South Korea; Department of Thoracic Surgery, Sejong General Hospital, 489-28 Hohyun-Ro, Sosa-Gu, Bucheon-Si, Kyoneggido 422-711 South Korea

**Keywords:** Double lumen tube, Hemoptysis, Aspiration, Lung separation, Thoracic surgery, Endobronchial cuff

## Abstract

**Background:**

Lung separation is essential for an emergency thoracic surgery for massive hemoptysis. When using a double lumen tube (DLT), a commonly adopted lung separation device during thoracic surgery, a water-tight seal of endobronchial cuff is crucial to prevent lung-to-lung aspiration of blood. In this study, we investigated the fluid sealing characteristics of the endobronchial cuff of a DLT and examined the effect of gel lubrication on the fluid leakage beyond the endobronchial cuff of DLT.

**Methods:**

An artificial tracheobronchial tree was intubated with a DLT. In the first phase of the study, the intra-cuff pressure of endobronchial cuff of DLT was set to 25, 50, or 100 cmH_2_O (n = 7, each), and the non-dependent bronchus was filled with 5 ml of water. Fluid leakage to the dependent bronchus beyond the endobronchial cuff was collected for 6 h. The time until leakage was first detected and the time until 100% leakage occurred were measured. In the second phase, the endobronchial cuff was coated with either saline (group C, n = 10) or lubricant gel (group GEL, n = 10), and the same parameters were measured.

**Results:**

In the first phase of the study, the times to first leakage and 100% leakage at an intra-cuff pressure of 25 cmH_2_O were 21.0 (7.0 - 59.0) sec and 3.0 (2.0 - 4.0) min, respectively. Higher intra-cuff (50 and 100 cmH_2_O) resulted in longer time for the first leakage and 100% leakage, but the duration was not long enough for clinical purpose. In the second phase, all the DLTs in group C showed 100% fluid leakage during the 6-hour period. In contrast, in group GEL, fluid leakage beyond the endobronchial cuff was detected only in 50% of the DLTs and none of the DLT showed 100% fluid leakage during the study. Among the DLTs which exhibited fluid leakage, the time to first leakage was 252.0 (171.0-305.0) min and the leakage volume at the end of the study period was 0.3〔0.0-1.8〕ml.

**Conclusions:**

Endobronchial cuff of DLT cannot prevent fluid leakage beyond the endobronchial cuff, but lubricant gel coating on the endobronchial cuff can effectively reduce the lung-to-lung aspiration.

## Introduction

Massive hemoptysis, traumatic or non-traumatic, is potentially lethal and has a high mortality rate [1,2]. Emergency surgery is an important treatment option [2-4] and sparing the non-bleeding lung from blood spill is critical in the perioperative period [1,5]. Patients undergoing thoracic surgery for hemoptysis are frequently placed in a lateral decubitus position to facilitate the surgical approach. In this position, the healthy lung is placed below the operative lung. Thus, preventing the gravity-driven drainage of blood from the operative lung into the healthy lung on the dependent side is an important clinical issue.

The double lumen tube (DLT) is the most commonly used lung isolation device during thoracic surgery [6]. When using the DLT, preventing leakage from one lung to the other depends on the sealing properties of the endobronchial cuff. It has been reported that endotracheal cuff of the modern single lumen endotracheal tubes (SLT) cannot reliably prevent fluid leakage [7-9]. The endobronchial cuff of the DLT has high-volume low-pressure (HVLP) characteristics, similar to those of endotracheal cuff of SLT [10-12]. As yet, the fluid sealing characteristics of endobronchial cuff of DLT in the lateral decubitus position have not been investigated in detail. In the first phase of our study, we investigated the sealing characteristics of endobronchial cuff of DLT in a lateral decubitus position using an artificial tracheobronchial tree.

For SLT, several methods have been suggested to improve the sealing characteristics of the endotracheal cuff. Of interest, the application of lubrication gel to the SLT endotracheal cuff has been shown to effectively reduce fluid leakage [13,14]. Thus, in the second phase of the study, we hypothesized that lubrication gel coating on the DLT endobronchial cuff could reduce fluid leakage past the cuff to reduce lung-to-lung spillage. The sealing characteristics of either lubrication gel-coated or saline-coated endobronchial cuffs were compared with respect to the volume and timing of fluid leakage past the cuff.

## Methods

Based on the previous reports [15], we simulated an artificial tracheobronchial tree with a 17-mm trachea that branched into 13-mm left and 16-mm right bronchi, and the study was conducted in two phases.

The first phase examined the fluid sealing characteristics of the DLT endobronchial cuff in the lateral decubitus position. The artificial tracheobronchial tree was intubated with a 35 Fr DLT (Broncho-cath^TM^, Mallinckrodt, Ireland). It was positioned horizontally, with one bronchus in the dependent position and the other bronchus in the non-dependent position. The intra-cuff pressure was set at 25, 50, or 100 cmH_2_O (groups 25, 50 and 100, respectively) using a cuff inflator (Cuff Pressure Manometer^TM^, Microcuff GmbH,Weinheim, Germany). After the cuff inflation, 5 ml of colored water were poured into the non-dependent side of the artificial bronchus to simulate blood or pus in the operative lung. Fluid leakage past the endobronchial cuff into the dependent bronchus was monitored. The leaked water was collected in a container placed below the open end of the dependent bronchus. The time until leakage was first detected and the time until 100% leakage occurred were measured. The time to 100% leakage was defined as the time point when 4.8 ml of fluid were collected in the container, because our pilot study revealed that 0.1- 0.2 ml of fluid was retained within the artificial tracheobronchial tree even when the cuff was completely deflated. The volume of fluid collected in the container was monitored for 6 h, and recorded by the minute during the first 15 min, at 30 min, and once per hour thereafter. Seven 35Fr DLTs were tested for each group.

In the second phase of the study, the effect of lubricating gel on the sealing characteristics of the cuff was investigated. The endobronchial cuff was lubricated with either saline or gel. In the saline-control group (group C; n = 10), the tube was dipped into bottle of saline before intubation. In the gel-lubrication group (group GEL; n = 10), 3 ml of water-soluble gel (K-Y Jelly^TM^, Johnson & Johnson, Korea) were applied onto a 10 × 10-cm four-ply gauze pad, and the pad was used to coat the cuff with gel. The artificial tracheobronchial tree was intubated with the DLT and positioned horizontally. The endobronchial cuff was inflated to pressure of 25 cmH_2_O and the volume of fluid leaking past the endobronchial cuff was measured for 6 h as in the first part of the study. The parameters measured in the first part of the study were also measured.

### Statistics

For the data from the first phase of the study, between- group comparisons were made using the Kruskal-Wallis test, with the Mann–Whitney *U* test as appropriate. The data from the second phase were compared using the Mann–Whitney *U* test. Value of *P* or the Bonferroni corrected *P* (*P* value multiplied by the number of comparisons) less than 0.05 were considered to indicate statistical significance. Data are presented as medians (interquatile).

## Results

**Figure 1:**
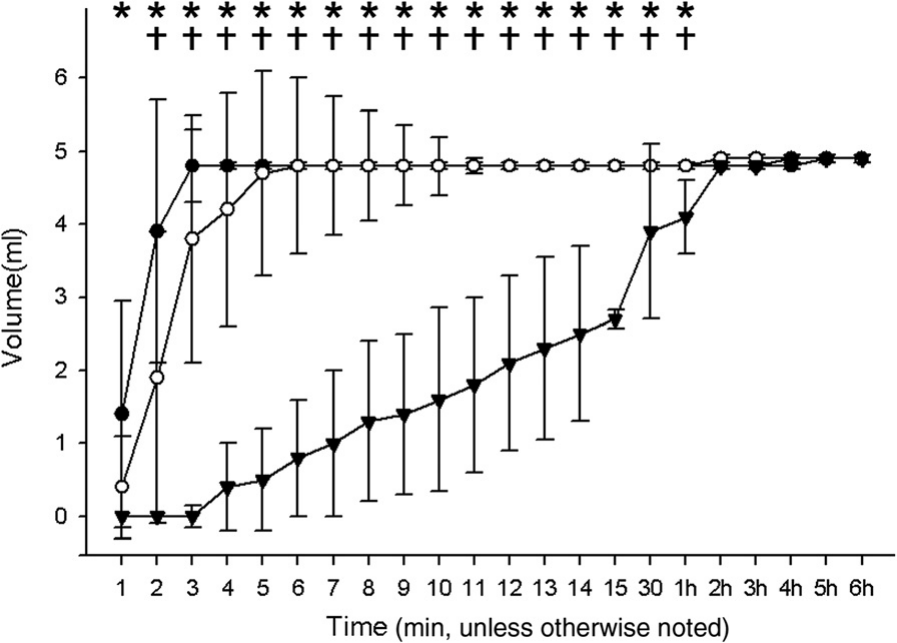
**Changes in fluid leakage volume over time at each intra-cuff pressure.** Data points are medians. Error bars represent interquartile ranges. Group 25: Intra-cuff pressure of 25 cm H_2_O (●); group 50: Intra-cuff pressure of 50 cm H_2_O (○); group 100: Intra-cuff pressure of 100 cm H_2_O (▼). * P < 0.008 among all three groups. † Corrected P < 0.020, group 100 *vs.* group 25 and group 100 *vs.* Group 50.

During the first phase of the study, the time until the first fluid leakage and the time until 100% leakage at each of the three intra-cuff pressures are presented in Table 1. The time to first leakage differed significantly among the groups. In pairwise comparisons, group 100 differed from groups 25 and 50, but no significant difference in time to first leakage was found between groups 25 and 50. The time to 100% leakage also differed significantly among the three groups. Compared with groups 25 and 50, group 100 exhibited a significantly longer time to 100% leakage. Groups 25 and 50 did not differ significantly with regard to time to 100% leakage. The volume of fluid that leaked over time at each intra-cuff pressure is presented in Figure 1. The volume of fluid that leaked differed significantly among the three groups from 1 min and 1 h. For the first hour, group 100 exhibited a significantly lower volume of leakage compared with groups 25 and 50, while groups 25 and 50 exhibited same amount of fluid leakage. By 2 h, 100% fluid leakage had occurred in all three groups, and there was no difference among them.

**Table 1:** **Time to first leakage and to 100% leakage of double-lumen tubes at each intra-cuff pressure**

**Group**	Time to first leakage (s)*	Corrected P-value*	**Time to 100% leakage (min)** ^**†**^	**Corrected P-value**
25	21.0 (7.0-59.0)	0.495^a^	3.0 (2.0-4.0)	0.786^a^
50	48.0 (32.0-78.0)	0.002^b^	6.0 (4.0-12.0)	0.002^b^
100	191 (125.0-260.0)	0.002^c^	85.0 (70.0-130.0)	0.002^c^

Data are presented as median (interquartile).

Group 25: Intra-cuff pressure of 25 cm H_2_O; group 50: Intra-cuff pressure of 50 cm H_2_O; group 100: Intra-cuff pressure of 100 cm H_2_O.

*P = 0.001 among all three groups.

^†^P < 0.001 among all three groups.

^a^Group 25 *vs*. group 50.

^b^Group 50 *vs*. group 100.

^c^Group 25 *vs.* group 100.

**Figure 2:**
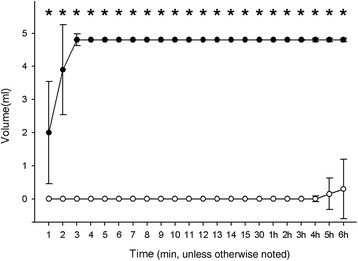
**Changes in fluid leakage volume over time based on gel lubrication of cuff.** Data points are medians. Error bars represent interquartile ranges. Saline group: endotracheal cuff was coated with saline (●); Gel group: endogracheal cuff was coated with lubrication gel (○).* P < 0.001 between the groups.

In the second phase of the study, the endobronchial cuff lubricated with gel had a significantly longer (*P* = 0.001) time to first leakage when compared with the saline control. The first fluid leakage was detected within 1 min [19.0 (9.8-34.3) s] in the group C. Among the 10 DLTs in the gel-coated group, only five showed fluid leakage during the 6-h study period, with a time to first fluid leakage of 252.0 (171.0-305.0) min. The time to 100% leakage in the group C was 3.0 (2.0-4.0) min, whereas none of the DLTs in group GEL exhibited 100% leakage during the study period. The median fluid leakage in the group GEL at the end of the study period was only 0.3 (0.0-1.8) ml. Figure 2 illustrates the significantly greater volume of fluid leakage in the group C compared with the group GEL at each time point during the study.

## Discussion

Our study showed that the DLT endobronchial cuff could not provide a water-tight seal against lung-to-lung fluid leakage in a lateral decubitus position but gel lubrication of the cuff effectively improved the sealing characteristics.

The modern polyvinylchloride–endotracheal cuff has HVLP characteristics [10]. An intra-cuff pressure of around of 25 cmH_2_O is recommended for HVLP cuff to avoid mucosal damage [16,17]. However, it has been well documented that this pressure cannot prevent fluid leakage around the HVLP endotracheal cuff [8,18]. The endobronchial cuff of DLT also has HVLP characteristics similar to those of the SLT endotracheal cuff [11,12], and our results show that in a lateral decubitus position, the DLT endobronchial cuff cannot provide a water tight seal under the recommended intra-cuff pressure of 25 cm H_2_O as that of endotracheal cuff of SLT. For the endotracheal cuff of the SLT, some studies have reported that higher intra-cuff pressures of 50–60 cmH_2_O can stop or reduce fluid leakage [7,9], whereas other groups have reported that an increase in intra-cuff pressure to 50 cmH_2_O is unable to prevent leakage [8]. During the present study, we examined the effect of pressure escalating on sealing characteristics of the endobronchial cuff of DLT by applying intra-cuff pressures of 25, 50, and 100 cmH_2_O to the endobronchial cuff. Compared with 25 and 50 cmH_2_O, an intra-cuff pressure of 100 cmH_2_O showed significantly longer times to both first leakage and 100% leakage. However, even at a pressure of 100 cm H_2_O, the times to first fluid leakage (median; 191 sec) and 100% leakage (median; 85 min) do not seem to be long enough for complicated thoracic surgery for hemoptysis which poses a high risk for lung-to-lung aspiration. In addition, the intra-cuff pressure of 100 cm H_2_O possesses a risk of airway injury [11,16,17]. Thus, despite the statistically beneficial results at 100 cm H_2_O, the clinical benefit of increasing the endobronchial cuff pressure above 25 cm H_2_O is doubtful.

Gel lubrication of the SLT cuff has been reported to prevent fluid leakage and reduce the risk for pulmonary aspiration [13]. The fluid leakage past the HLVP cuff occurs because the diameter of the high-volume cuff exceeds the diameter of the tracheal lumen. Upon inflation, numerous longitudinal folds are formed on the surface of the cuff and they permit fluid leakage around the cuff [7-9]. By using lubricant gel, these folds can be effectively sealed and fluid leakage past the HVLP cuff can be prohibited [13]. In agreement with previous study regarding endotracheal cuffs, the use of gel lubricants effectively reduced fluid leakage past the endobronchial cuff in this study. Gel lubricant has been widely applied to airway management in clinical practice such as facilitating endotracheal intubation [19,20], flexible bronchoscopy insertion [21], laryngeal mask airway insertion [22], and endotracheal stylet use [23]. In this respect, gel lubrication of the endobronchial cuff could be safely applied in clinical practice to reduce lung-to-lung contamination via a DLT in patients undergoing emergency thoracic surgery for hemoptysis.

## Conclusions

To sum up, the endobronchial cuff of a DLT cannot provide an adequately water-tight seal against lung-to-lung contamination even with higher intra-cuff pressure than the recommended range, and gel lubrication of the endobronchial cuff effectively improves its sealing characteristics under a recommended intra-cuff pressure. We recommend consideration of the application of gel lubrication on the endobronchial cuff of DLT to reduce lung-to-lung aspiration during emergency thoracic surgery for hemoptysis.
